# Factors associated with cyber-victimization among immigrants and non-immigrants in Canada: a cross-sectional nationally-representative study

**DOI:** 10.1186/s12889-020-09492-w

**Published:** 2020-10-16

**Authors:** Kathleen S. Kenny, Lisa Merry, Douglas A. Brownbridge, Marcelo L. Urquia

**Affiliations:** 1 Manitoba Centre for Health Policy, University of Manitoba, 408-727 McDermot Ave, Winnipeg, Manitoba R3E 3P5 Canada; 2 Department of Community Health Sciences, Rady Faculty of Health Sciences, University of Manitoba, Winnipeg, Canada; 3 Faculty of Nursing, University of Montreal, Montreal, Canada; 4 Dalla Lana School of Public Health, Faculty of Medicine, University of Toronto, Toronto, ON Canada

**Keywords:** Cyberbullying, Immigrants, Gender, Victimization, Neighborhood/place

## Abstract

**Objectives:**

There is a paucity of research on patterns of cyber-victimization in minority groups, including immigrants. This study aimed to identify individual, interpersonal and contextual characteristics associated with cyber-victimization among immigrants and non-immigrants.

**Methods:**

We drew on nationally representative data from adolescents and adults in the Canadian General Social Survey on victimization (2014). We used multivariable logistic regression to identify potential factors associated with cyber-victimization in the last 12 months, stratified by immigrant status and sex.

**Results:**

Among 27,425 survey respondents, the weighted prevalence of cyber-victimization in the last 12 months was 2.1% among immigrants and 2.3% among non-immigrants. Cyber-victimization rates differed significantly by sex among immigrants (2.8% for males vs. 1.4% for females), but not among non-immigrants (2.1% for males vs. 2.4% for females). While most other factors associated with cyber-victimization were similar for immigrants and non-immigrants, there were pronounced associations of past child maltreatment (adjusted prevalence odds ratio [aPOR] 4.85, 95% confidence interval [CI] 2.76, 8.52) and residence in an unwelcoming neighbourhood (aPOR 5.08, 95% CI 2.44, 10.55) with cyber-victimization among immigrants that were diminished or absent among non-immigrants. Additionally, sex-stratified analyses among immigrants showed cyber-victimization to be strongly associated with having a mental health condition (aPOR 3.50, 95% CI 1.36, 8.97) among immigrant males only, and with perceived discrimination (aPOR 4.08, 95% CI 1.65, 10.08), as well as being under 24 years old (aPOR 3.24, 95% CI 1.09, 9.60) among immigrant females.

**Conclusions:**

Immigration status and sex were differentially associated with cyber-victimization. Findings support the salience of a social-ecological perspective and gender-stratified analyses to better elucidate complex pathways linking cyber-victimization to potential gender-based health inequities among immigrants.

## Introduction

Cyberbullying is the use of computers, cell phones or other technological devices to deliberately threaten, abuse, or intimidate an individual or a group of individuals [1, 2]. This form of bullying is posited by some as more insidious than traditional in-person bullying by allowing for anonymity of the perpetrator(s) and instant reach to broad audiences [3, 4]. Previous research has identified cyber-victimization as a growing public health problem in young people, associated with increased depression [4], suicidal ideation [5], substance use [6], poorer physical health [7], and lower self-esteem [3].

As individuals increasingly spend more time on-line, considerable attention has been placed on understanding and mitigating risks associated with cyberbullying. In two meta-analyses focused on young people, those who were cyber-bullied were identified as more likely to engage in frequent internet activities, experience off-line bullying, have low self-esteem, report depression and anxiety, and use drugs and alcohol [1, 8]. This literature, however, also points to challenges in estimating causal pathways, since psychological, physical and social problems are identified as both risk factors and sequelae of cyberbullying, presenting limitations to temporal ordering [1]. Furthermore, a knowledge gap has been identified in understanding how various social characteristics, such as race, ethnicity, class, gender, immigrant status, and/or sexual orientation, may differentially influence cyberbullying risk [9–11]. Immigrants in particular can face a host of challenges related to the resettlement process, which previous research shows can heighten the risk of ‘in-person’ bullying [12, 13], thus lending plausibility to the likelihood that being an immigrant may also increase the risk of cyber-victimization. Additionally, immigrants more often maintain social ties within immigrant networks [14] and across borders [15], which may also contribute to cyberbullying. Given these unique circumstances of immigrants, understanding the potential risk factors of cyberbullying in this population constitutes an important step in addressing implications for immigrant health and tailoring resources for prevention.

There is a paucity of literature on cyberbullying among immigrants and it remains unclear what factors contribute to prevalence in this population. In one of the only studies comparing cyber-bullying rates of immigrant and US-born youth, immigrants were 2.27 times (95% confidence interval [CI] 1.62, 3.39) more likely to report cyber-victimization than non-immigrants [11]. Similarly, results from studies focused on ‘traditional’ in-person bullying also indicated a higher burden of bullying among immigrant youth [11, 16–18], and though existing knowledge of potential mechanisms is limited, several intra- and inter-personal factors associated with ‘traditional’ bullying have been identified. These factors include having few close friends, dissatisfaction with family relationships, and loneliness, all of which are also known factors associated with adapting to a new country [11, 19]. Studies further show that immigrant youth were more likely than non-immigrants to be bullied on religious or racial grounds [20, 21], an observation that points to the potential role of anti-immigrant sentiment in host countries as an additional mechanism underlying bullying risk in this population; and one that may also extend to experiences of cyber-bullying [18, 22]. Further, while prior research supports the role of immigrant networks and transnational networks in buffering the socially disruptive event of migration [14, 15], there has been no empirical studies examining whether network contacts in the receiving country and/or country of origin may also be sources of cyberbullying.

Gender is a prominent factor among socio-demographic variables examined in studies of cyber-victimization in immigrant populations. Since negative health outcomes (i.e., depression, anxiety, low self-esteem, lower-self reported physical health) associated with resettlement stress are shown to differ by immigrant group, the context of migration, and further, within-group, by age and gender [23, 24], it is likely that cyberbullying rates may also vary according to gender. While the majority of previous studies have not stratified analyses by gender, thus precluding interpretation of gender differences beyond the descriptive level, findings from one study indicated that young immigrant males faced an almost two-fold greater likelihood of cyber-victimization (adjusted odds ratio [aOR] 2.80, 95% CI 1.75, 4.50) than immigrant females (aOR 1.51, 95% CI 0.85, 2.68) [11]. This study, however, was limited by its use of US-born males and females as reference groups, and thus did not investigate the extent of an intra-immigrant gender gap. By contrast, among young people in the general population, there has been extensive examination of gender patterns that has largely reported inconsistent results, with some studies showing females to be more at risk of being victimized [7, 25], and other showing no gender-related differences [26–28].

Considerable gaps in the literature are also evident in understanding the relationship between age and cyberbullying in immigrants, and no prior studies have yet examined age-related patterns. Looking more broadly at the general population, results have shown no association between age and cyber-victimization among youth of different age groups, while pointing to a weak positive association of older youth with cyberbullying perpetration [1]. On this parameter it is notable that since the vast majority of previous studies on cyberbullying have almost exclusively focused on youth in school settings, there has been far less focus on cyber-victimization among older people [29]. Several studies of both college students [30, 31] and adults in the workplace [32–34], however, suggest a broad reach of this phenomenon, justifying the need for more empirical understanding of the breadth and scope of cyber-victimization across the lifespan, including among older immigrant populations.

With the growing ubiquity of internet use, the unique challenges of the resettlement process, and rising anti-immigrant policies [35, 36], it is also very plausible that the landscape of cyberbullying among immigrants may be influenced by the local neighbourhood microcosm, including local anti-immigrant sentiment, discrimination, and violence. In one of the only known studies to date examining neighbourhood-level influences on cyberbullying perpetration, Khoury Kassabri et al. (2016) found an association between neighbourhood violence and cyberbullying perpetration among Arab minority youth residing in Israel [37]. Though no study to date has examined the relationships between neighbourhood-level factors and cyberbullying specifically among immigrants, research on links between discrimination and the resettlement process with mental health morbidity [20], suggest neighbourhood/place entail important dimensions of the immigrant experience that could also assist in explaining susceptibility to cyber-victimization and related health impacts.

In the present study, we used data from a nationally-representative sample of Canada, a country with a high proportion of immigrants (21%) that is second only to Australia (27%) [38]. Adopting a contextual and comparative approach, we drew on Bronfenbrenner’s (1979) social-ecological model to help understand to what extent multiple interacting levels of the social ecology, including other forms of victimization, are associated with cyberbullying among immigrants and non-immigrants [39]. With this theoretical orientation, justified by calls in the literature for a broader view of this phenomenon [1, 37, 40], we aimed to account for socio-demographic variables at the individual, interpersonal and neighbourhood-levels that prior research has shown as potential risk and protective factors associated with cyberbullying. To compare patterns of cyberbullying according to immigrant status, we first examined the prevalence of factors associated with being cyber-bullied in the last 12 months stratified by immigrant status (i.e., immigrant to Canada vs. non-immigrant). Based on the prominence of gender in prior cyberbullying analyses, we then examined prevalence of factors associated with cyberbullying stratified by immigrant status and sex (male/female).

## Methods

### Data source and sample

This study used data collected from January to December 2014 by Statistics Canada’s Canadian General Social Survey (GSS), Cycle 28 on Victimization [41]. The main objective of the GSS on Canadians’ Safety (Victimization) was to better understand how Canadians perceive crime and the justice system and to capture information on experiences of victimization. In this vein, surveys comprised questions about the nature and extent of respondents’ victimization, including experiences of cyberbullying. This telephone population-based survey included a random sample of non-institutionalized persons aged 15 years and older living in the 10 Canadian provinces. The sample was constructed through a complex, multi-stage sampling design to obtain representative coverage of Canadian households with a telephone number. All households in Canada with telephone numbers were ascertained through a list of registered phone numbers (both land-line and cellular numbers) and a registry of all dwellings in the 10 provinces. Once a household was selected and contacted by phone, an individual 15 years or older was randomly selected to complete the survey. An oversample of immigrants and youth was added to the 2014 GSS for a more detailed analysis of these groups.

Survey responses were obtained by computer-assisted telephone interviews conducted in the Canadian official languages (English or French) of the respondent’s choice. Households without telephones (approximately 1% of the target population) were not captured. The response rate was 53%. Total non-response was handled by adjusting the weight of households who responded to the survey to compensate for those who did not respond. Non-respondents included people who refused to participate, could not be reached, or could not speak English or French. The sample size was 33,127 respondents. Since cyberbullying was the focus of this study, we then excluded about 16% of respondents who reported either not using the internet in the past 5 years, did not know, or refused to answer, as well as < 1% of respondents who refused to answer questions related to cyberbullying and those for whom immigrant status could not be determined. The final analytic sample included 27,425 individuals.

### Measures

Cyber-victimization in the last 12 months: Cyber-victimization was assessed according to responses to a series of 5 questions asking whether in the last 5 years a respondent ever had any threatening or aggressive emails/messages; threatening or aggressive comments directed at them via group emails/messages or internet postings; embarrassing/threatening pictures posted of them; embarrassing/threatening information posted by someone pretending to be them; and any other type of cyber stalking/bullying. If a respondent answered affirmatively to any of these questions they were asked a follow-up question about whether any of these experiences occurred in the past 12 months. Cyber-victimization was thus constructed as a dichotomous variable specified as any cyber-victimization reported in the last 12 months versus none.

Immigrant status: The key independent variable of interest was immigrant status. Respondents were classified as immigrants if they reported being born outside Canada and obtained legal permanent residency/citizenship. Respondents who did not report permanent residency, but reported being born outside of Canada and provided a year for when they first came to live in Canada, were also considered immigrants, assessed as likely to have arrived in Canada as asylum seekers, students or temporary workers.

Explanatory factors were identified based on the literature and available data. Since a majority of previous research on cyberbullying involvement has been limited to individual-level factors, this study incorporated a broader social-ecological emphasis, including attention to how individual, interpersonal and neighbourhood factors may explain patterns of cyber-victimization. Individual characteristics included sex (male vs. female), age (15–24, 25–34, 35–44, 45+ years), education (high school completion vs. incomplete), visible minority status (visible minority vs. non-visible minority) based on the definition in the Canadian Census, excludes Indigenous peoples and consists mainly of the following groups: South Asian, Chinese, Black, Fillipino, Latin American, Arab, Southeast Asian, West Asian, Korean and Japanese, annual household income (under $20,000 vs. $20,000 or above), disability status (any vision, hearing, physical, learning, mental/psychological or other disability vs. none), mental health (any emotional, psychological or mental health condition vs. none), and alcohol use (categories: 5 or more drinks on same occasion in past month, fewer than 5 drinks on same occasion in past month, abstained from alcohol in past month). Interpersonal characteristics included history of childhood victimization (any physical or sexual abuse before age 15 years vs. none), number of close friends or relatives (0–5 friends/relatives, 6–10, more than 10), intimate partner violence (IPV) in last 5 years (any physical or sexual violence by spouse, ex-spouse or dating partner), and any discrimination experienced in last 5 years (victim of any discrimination based on sex, ethnicity/culture, race/skin colour, physical appearance, religion, sexual orientation, age, physical or mental disability, language or other vs. none). Neighbourhood-level characteristics included respondents’ perspectives of how welcoming their neighbourhood is (residing in an unwelcoming community vs. residing in a welcoming community), neighbourhood discrimination (people attacked in neighbourhood based on skin colour, ethnic origin or religion vs. none), and neighbourhood trust (dichotomized based on an ordinal variable with high/moderate trust in neighbours as referent category and low/no trust in neighbours as index category) [42].

### Statistical analyses

Descriptive statistics were calculated for all variables and stratified separately by cyber-victimization and immigrant status. Subsequently, we estimated the odds of cyber-victimization with each covariate using unadjusted logistic regression models and applying bootstrapped sampling weights to account for complexity of the survey design and produce nationally representative estimates. To obtain estimates stratified by immigrant status and, further, by immigrant status and sex, six multivariable logistic models were constructed: Model 1: immigrants-only; Model 2: non-immigrants only; Model 3: immigrant females; Model 4: immigrant males; Model 5: non-immigrant females; and Model 6: non-immigrant males. Explanatory variables for final models were selected based on Hosmer et al.’s (2013) purposeful selection of covariates criteria [43]. To avoid over-adjustment of multivariable models, multicollinearity was assessed using variance inflation factors and Pearson correlation for each variable pair. All data management, programming and analyses were performed using SAS® version 9.4. The study obtained ethics approval from the Research Ethics Board at the University of Manitoba (Protocol reference: H2018:438 (HS22337)).

## Results

Using a weighted sample of 27,425 respondents with bootstrapped sampling weights to make the data nationally-representative of the Canadian population, the prevalence rate of cyber-victimization in the last 12 months was 2.1% among immigrants and 2.3% among non-immigrants, respectively (Table 1). In unadjusted analyses, both immigrants and non-immigrants who were cyberbullied tended to be single, to be living with a disability, to have a mental health condition, to have a history of child maltreatment, to have experienced discrimination, and to reside in neighbourhoods perceived as unwelcoming or discriminatory. Additionally, immigrants exposed to cyber-victimization were more likely to be a non-visible minority than those unexposed, while among non-immigrants, the exposed were more likely to be younger, to have lower incomes, to have not completed high school, to have a history of IPV, to have fewer close friends/ relatives, and to live in a neighbourhood characterized by higher distrust of neighbours. Further, in unadjusted analyses, cyber-victimized immigrants were significantly more likely than non-immigrants to be male (POR 0.56, 95% CI 0.37, 0.85) (*p*-value for interaction of immigrant status with sex: 0.008), to have experienced prior child maltreatment (POR 0.52, 95% CI 0.33, 0.80), and to have a larger network of close friends and relatives (POR 0.59, 95% CI 0.38, 0.92) (Table 2).

**Table 1:** Weighted Sample Characteristics Stratified by Immigrant Status and Cyberbullying, General Social Survey, Canada, 2014

	Immigrants (unweighted *n* = 6273)		Non-Immigrants (unweighted *n* = 21,152)	
	Cyberbullying (%)	No Cyberbullying (%)	Cyberbullying (%)	No Cyberbullying (%)
Cyberbullying in last 12 months	2.1	97.9	2.3	97.7
**Individual-level**				
Sex				
Male	67.2	50.1	45.8	49.6
Female	32.8	49.9	54.2	50.4
Age				
15–24	16.7	10.5	28.7	19.4
25–34	24.4	20.0	21.6	18.6
35–44	17.9	21.2	17.4	16.5
45+ years	41.0	48.3	32.3	45.5
Household income				
Under $20,000	49.3	41.8	39.9	31.1
$20,000 +	46.2	55.6	56.6	66.7
Unknown	4.5	2.6	3.5	2.2
Educational attainment				
High school not completed	9.6	6.5	17.1	12.2
High school or more	90.4	92.9	82.8	87.3
Missing		0.6	0.1	0.5
Marital status				
Married or common-law	48.4	69.8	40.7	59.6
Widowed/separated/divorced	17.4	7.2	9.3	8.7
Single, never married	34.2	23.0	49.9	31.6
Missing			0.1	0.1
Visible minority				
Yes	43.0	59.8	6.0	5.5
No	55.7	38.8	92.7	94.1
Missing	1.3	1.4	1.3	0.4
Disability				
Yes	33.0	18.6	39.6	23.8
No	67.0	80.6	59.1	75.8
Missing		0.8	1.3	0.4
Mental health condition				
Yes	30.5	8.7	37.9	14.6
No	69.5	90.9	59.6	85.1
Missing		0.4	2.5	0.3
Alcohol use in past month				
High	15.3	15.1	38.3	31.8
Low	47.7	40.6	33.4	42.1
None	37.0	43.8	27.7	25.3
Missing		0.5	0.6	0.8
**Interpersonal-level**				
History of childhood victimization				
Yes	72.5	29.5	50.4	31.4
No	26.8	66.7	47.8	66.8
Missing	0.7	3.8	1.8	1.8
Number of close friends/relatives				
0–5	32.8	42.2	36.6	30.3
6 to 10	39.0	30.0	34.5	35.1
More than 10	26.7	25.3	26.7	33.3
Missing	1.5	2.5	2.2	1.3
Any IPV				
Yes	6.8	3.1	14.0	4.3
No	92.1	95.9	85.2	95.2
Missing	1.1	1.0	0.8	0.5
Perceived discrimination				
Yes	44.5	17.5	30.7	12.7
No	55.5	82.4	69.2	87.2
Missing		0.1	0.1	0.1
**Neighbourhood-level**				
Unwelcoming neighbourhood				
No	69.7	91.9	88.1	93.2
Yes	26.5	5.9	11.6	5.5
Missing	3.8	2.2	0.3	1.3
Neighbourhood discrimination				
Yes	21.9	7.2	16.2	6.1
No	75.5	90.9	82.1	92.3
Missing	2.6	1.9	1.7	1.6
Neighbours can be trusted				
High/moderate trust	54.0	40.7	47.5	33.5
Low/no trust	42.4	55.7	52.2	65.5
Missing	3.6	2.6	0.3	1.0

**Table 2:** Bivariable Associations of Individual, Interpersonal and Neighbourhood Factors with Cyberbullying

	Immigrants			Non-Immigrants			Immigrants vs. Non-immigrants	
	%	POR	95% CI	%	POR	95% CI	POR	95% CI
Cyberbullying in the last 12 months	2.1			2.3				
**Individual-level**								
Sex								
Male	2.8	2.05	1.17, 3.58	2.1	0.86	0.66, 1.12	1.34	0.84, 2.14
Female	1.4	1.00	ref.	2.4	1.00	ref.	0.56	0.37, 0.85
Age								
15–24 years	3.3	1.86	0.81, 4.29	3.3	2.08	1.51, 2.87	0.98	0.53, 1.82
25–34 years	2.5	1.44	0.65, 3.20	2.6	1.63	1.13, 2.34	0.97	0.49, 1.90
35–44 years	1.8	0.99	0.47, 2.09	2.4	1.48	1.06, 2.06	0.74	0.44, 1.25
45 years +	1.8	1.00	ref.	1.6	1.00	ref.	1.10	0.58, 2.07
Household income								
Under $20,000	2.5	2.11	0.94, 4.74	2.9	1.88	1.17, 3.01	1.01	0.44, 2.33
Unknown	3.6	1.42	0.79, 2.54	3.6	1.51	1.15, 2.00	0.84	0.49, 1.46
$20,000 +	1.7	1.00	ref.	1.9	1.00	ref.	0.90	0.61, 1.33
Educational attainment								
High school not completed	3.1	1.51	0.54, 4.26	3.2	1.48	1.02, 2.13	0.97	0.35, 2.63
High school or more	2.0	1.00	ref.	2.2	1.00	ref.	0.94	0.65, 1.37
Marital status								
Married or common law	1.5	1.00	ref.	1.6	1.00	ref.	0.93	0.62, 1.40
Widowed, separated, divorced	4.9	3.48	0.87, 13.98	2.4	1.55	1.11, 2.18	2.09	0.53, 8.31
Single, never married	3.1	2.14	1.20, 3.83	3.5	2.31	1.75, 3.05	0.87	0.53, 1.41
Visible minority								
Yes	1.5	0.50	0.28, 0.91	2.5	1.11	0.56, 2.19	0.61	0.28, 1.30
No	3.0	1.00	ref.	2.2	1.00	ref.	1.34	0.81, 2.21
Disability								
Yes	3.7	2.14	1.04, 4.42	3.7	2.14	1.65, 2.76	0.98	0.48, 1.99
No	1.7	1.00	ref.	1.8	1.00	ref.	0.98	0.69, 1.40
Mental health condition								
Yes	7.0	4.61	2.09, 10.18	5.7	3.70	2.83, 4.84	1.25	0.58, 2.72
No	1.6	1.00	ref.	1.6	1.00	ref.	1.00	0.72, 1.40
Alcohol use								
High	2.1	1.20	0.51, 2.80	2.7	1.10	0.79, 1.54	0.77	0.39, 1.52
Low	2.4	1.39	0.89, 2.81	1.8	0.73	0.53, 1.00	1.36	0.90, 2.06
None	1.8	1.00	ref.	2.5	1.00	ref.	0.71	0.37, 1.37
**Interpersonal-level**								
History of childhood victimization								
Yes	5.0	6.12	3.56, 10.52	3.6	2.24	1.74, 2.89	1.41	0.91, 2.17
No	0.9	1.00	ref.	1.6	1.00	ref.	0.52	0.33, 0.80
Number of close friends/relatives								
0–5	1.6	0.74	0.30, 1.83	2.7	1.51	1.14, 2.00	0.59	0.38, 0.92
6 to 10	2.7	1.23	0.47, 3.25	2.2	1.23	0.90, 1.68	1.21	0.72, 2.04
More than 10	2.2	1.00	ref.	1.8	1.00	ref.	1.21	0.50, 2.96
Any IPV								
Yes	4.5	2.28	0.94, 5.53	7.0	3.61	2.55, 5.11	0.62	0.26, 1.51
No	2.0	1.00	ref.	2.0	1.00	ref.	0.99	0.69, 1.41
Perceived discrimination								
Yes	5.2	3.78	2.02, 7.09	5.3	3.04	2.28, 4.04	0.97	0.53, 1.78
No	1.4	1.00	ref.	1.8	1.00	ref.	0.78	0.54, 1.12
**Neighbourhood-level**								
Unwelcoming neighbourhood								
Yes	8.7	5.91	2.34, 14.92	4.7	2.26	1.57, 3.25	0.74	0.53, 1.03
No	1.6	1.00	ref.	2.1	1.00	ref.	1.93	0.77, 4.86
Neighbourhood discrimination								
Yes	6.1	3.67	1.31, 10.28	5.8	3.00	2.01, 4.47	1.05	0.37, 2.99
No	1.7	1.00	ref.	2	1.00	ref.	0.86	0.62, 1.18
Neighbourhood trust								
High/moderate trust	2.8	1.00	ref.	3.2	1.00	ref.	0.86	0.52, 1.43
Low/no trust	1.6	1.77	0.95, 3.29	1.8	1.78	1.37, 2.31	0.86	0.57, 1.31
n (unweighted)			6273			21,152		

Abbreviations: *POR* prevalence odds ratio, *95% CI* 95% confidence interval

Note: Prevalence and POR estimates are weighted

### Cyber-victimization and immigration status

In multivariable adjusted models stratified by immigration status (Table 3), factors positively associated with cyber-victimization among immigrants (Model 1) included being male, being single, reporting a mental health condition, having a history of child maltreatment, and residing in an unwelcoming neighbourhood. Results for non-immigrants (Model 2) showed a different pattern, where being male was not associated with cyber-victimization, associations with child maltreatment, mental health and single status were less pronounced, and other factors, including past IPV, being a victim of discrimination and neighbourhood discrimination, conferred additional explanatory power. Cybervictimization was not associated with visible minority status in either group.

**Table 3:** Multivariable Adjusted Prevalence Odds Ratios of Factors Associated with Cyberbullying by Immigrant Status

	Model 1: Immigrants		Model 2: Non-immigrants	
	aPOR	95% CI	aPOR	95% CI
**Individual-level**				
Sex (male)	1.93	1.06, 3.50		
Marital status: Married/common law	1.00	ref.	1.00	ref.
Single (never married)	2.04	1.10, 3.79	1.87	1.38, 2.52
Divorced, separated, widowed	2.68	0.93, 7.70	1.27	0.87, 1.84
Mental health condition	2.99	1.54, 5.81	2.58	1.86, 3.57
**Interpersonal-level**				
History of child maltreatment	4.85	2.76, 8.52	1.59	1.22, 2.09
Any IPV			2.04	1.36, 3.06
Perceived discrimination			1.72	1.20, 2.48
**Neighbourhood-level**				
Unwelcoming neighbourhood	5.08	2.44, 10.55		
Neighbourhood discrimination			1.83	1.15, 2.92

Abbreviations: *aPOR* adjusted prevalence odds ratio, *95% CI* 95% confidence interval

Models are adjusted for the number of predictors included in the model

All estimates are weighted using bootstrapped sampling weights

Empty cells indicate that variables were dropped from final models

### Cyber-victimization, immigrant status and sex

In multivariable adjusted models stratified by immigrant status and sex (Table 4), a history of child maltreatment and residing in an unwelcoming neighbourhood remained stable predictors of cyber-victimization among both immigrant females and males. Differences between immigrant females (Model 3) and immigrant males (Model 4) included having a mental health condition, which was associated with cyber-victimization among immigrant males only, and being of younger age, consuming low levels of alcohol (vs. abstaining), and being a victim of discrimination, which were uniquely associated with cyber-victimization among female counterparts. Among non-immigrants, while having a mental health condition, being a victim of discrimination, and past IPV remained strong predictors across sexes, non-immigrant females who were cyber-victimized were more likely to be of younger age and to have less than a high school education (Model 5), where by contrast among non-immigrant males (Model 6), cyber-victimization was positively associated with being single, having a history of child maltreatment, and residing in a neighbourhood perceived as discriminatory.

**Table 4:** Multivariable Adjusted Prevalence Odds Ratios of Factors Associated with Cyberbullying by Immigrant Status and Sex

	Immigrants				Non-immigrants			
	Model 3: Female		Model 4: Male		Model 5: Female		Model 6: Male	
	aPOR	95% CI	aPOR	95% CI	aPOR	95% CI	aPOR	95% CI
**Individual-level**								
Age: 45+ years	1.00	ref.			1.00	ref.		
15–24 years	3.24	1.09, 9.60			1.63	1.04, 2.57		
25–44 years	2.76	1.11, 6.83			1.33	0.91, 1.94		
Marital status: Married/common law			1.00	ref.			1.00	ref.
Single (never married)			2.17	0.91, 5.15			2.06	1.33, 3.20
Divorced, separated, widowed			4.02	0.70, 23.20			0.89	0.43, 1.85
Education (high school completed)					1.80	1.09, 2.97		
Mental health condition			3.50	1.36, 8.97	2.93	1.99, 4.32	2.28	1.37, 3.78
Alcohol use in past month: None	1.00	ref.						
High	2.22	0.65, 7.58						
Low	2.70	1.07, 6.79						
**Interpersonal-level**								
History of child maltreatment	5.12	1.93, 13.58	4.18	1.97, 8.86			2.10	1.38, 3.19
Number of close friends/relatives: More than 10							1.00	ref.
0–5							1.29	0.78, 2.12
6 to 10							1.23	0.72, 2.10
Any IPV					1.70	1.02, 2.86	2.98	1.60, 5.56
Perceived discrimination	4.08	1.65, 10.08			1.71	1.11, 2.65	2.12	1.22, 3.68
**Neighbourhood-level**								
Unwelcoming neighbourhood	3.60	1.44, 9.02	5.46	1.95, 15.31				
Neighbourhood discrimination							2.38	1.22, 4.65
n (unweighted)		2891		2672		10,651		8785

Abbreviations: *aPOR* adjusted prevalence odds ratio, *95% CI* 95% confidence interval

Models are adjusted for the number of predictors included in the model

Due to stratification by gender, categories of age and household income had to be collapsed due to low cell count

All estimates are weighted using bootstrapped sampling weights

Empty cells indicate that variables were dropped from final models

## Discussion

In a large nationally-representative sample of people aged 15 years and older living in Canada, we found a similar prevalence of cyber-victimization among immigrants and non-immigrants. Significantly, while most factors associated with cyber-victimization were similar between the two groups, we found important differences both according to immigrant status and further in subgroups defined by sex.

### Cyber-victimization and immigrant status

At the individual-level, cyber-victimization was positively associated with being male among immigrants, and was not associated with being male (or female) among Canadian-born counterparts. Results also showed a strong association between cyber-victimization and having a history of child maltreatment, which was most notable among immigrants. Additionally, at the interpersonal-level, while we found that having a history of IPV was strongly associated with cyber-victimization among non-immigrants, there was no evidence of an association among immigrants. Further, at the neighbourhood-level, we found that perceiving to reside in an unwelcoming neighbourhood, rather than individual or interpersonal factors, was associated with the strongest association to cyber-victimization for immigrants, and did not find evidence of a similar pattern among non-immigrants.

### Cyber-victimization, immigrant status and sex

At the individual-level, among immigrant females, we found those who were cyber-victimized tended to have significantly higher odds of being younger, a pattern that was also observed, though to a lesser degree, among non-immigrant females. Further, results showed a positive association between cyber-victimization and having a mental health condition among immigrant males that was also evident among non-immigrant males and females, yet notably absent among immigrant females. Additionally at the interpersonal-level, we found evidence of a strong association between cyber-victimization and perceived discrimination among immigrant females, which was far more modest among non-immigrants groups, and absent among immigrant males.

The link between cybervictimization and being male among immigrants suggests a differential vulnerability between immigrant men and women that is consistent with findings in one previous study of school-age youth that similarly showed immigrant males to have higher odds of both cyber and other forms of bullying than females [11]. It is possible that this pattern speaks to processes by which resettlement experiences may differently contribute to the risk of cyberbullying for immigrant males compared to females. One explanation could be that the increase in migration to Canada from countries of origin where gender norms are more likely to differ from those in Canada, is creating a heightened vulnerability for immigrant men characterized by a combination of stressful conditions, including less social acceptance and emotional distress, which, like ‘traditional’ bullying victimization [44] may also influence risk of cyberbullying. While the mechanisms through which mental health is linked to cyber-bullying remain a matter of some debate [1, 8], our sex-stratified results among immigrants showing a link between mental health and cyber-bullying among males only, may further help explain why gendered differences in the resettlement process could make immigrant men more vulnerable to cyber-victimization. Sex-stratified-analyses within immigrants also identified increased risk for cyberbullying among younger immigrant females that was not observed among male counterparts. Indeed, while being of younger age was also a risk factor among non-immigrant females, the appreciable association among immigrant females suggests that the developmental stage of adolescence may be a particularly sensitive period of heightened cyber-related risk for this group, of which the extent and impact require further investigation.

The high degree of overlap between cyber-victimization and other forms of interpersonal victimization is consistent with prior literature on young people in the general population [45, 46], which suggests exposure to one form of victimization is associated with exposure to other types of victimization [47]. Notably, immigrants who reported a history of child maltreatment were more than three times as likely to experience cyber-victimization than those who did not experience child maltreatment. This marks a potential extension of the literature on links between child maltreatment and cyber-victimization, and further, sheds light on another adversity that may contribute to prevalence of cyberbullying risk among immigrants [48]. Since prior literature strongly highlights the role of positive family support/relationships as protective against cyber-victimization [49, 50], it may be that the higher likelihood of cyber harms among immigrants with a history of maltreatment is linked to the role of family in the migration context. In this environment of elevated reliance on family, the effects of child maltreatment, including higher dissatisfaction with family relationships, lower self-esteem, and social isolation [51], could be evenmore jeopardizing for immigrants, and thus also contribute to increased likelihood of other forms of victimization, including cyber-victimization [11, 16–18]. Furthermore, the positive association between cyber-victimization and perceived discrimination, which was most pronounced for immigrant females, could also indicate that poly-victimization, inclusive of cyber-bullying, is more prevalent among immigrants compared to non-immigrants [52] The pattern of poly-victimization, however, did not include IPV, where we found a positive association between cybervictimization and IPV among non-immigrants that did not extend to immigrants. While we find this latter result counterintuitive, we suspect it could relate to the under-identification of IPV reporting among immigrants [53].

Our results also point to an important role of perceived neighbourhood characteristics in explaining patterns of cyberbullying, and show these links to be more strongly and consistently observed among immigrants than non-immigrants. Most significantly, we found that the perception of residing in an unwelcoming neighbourhood was very strongly associated with cyber-victimization across all immigrants regardless of sex, but found no discernible association among non-immigrants, for whom perceived neighbourhood-level discrimination was more often associated with cyber-victimization. These results, and recent research [54], encourage examination of the ways cyber-space intersects with neighbourhood/place. Of particular importance, the apparent divergence between experiences of neighbourhood ‘welcome’ and ‘discrimination’ in explaining cyberbullying risk among immigrants and non-immigrants could indicate that cohesion within a neighbourhood may be associated with risk through separate pathways based on immigrant status. For example, an individual’s perception of an ‘unwelcoming’ neighbourhood may be a more adequate proxy than ‘discrimination’ in explaining the link between inclusion/exclusion and risk of cyber-victimization for immigrants, and could reflect how rising anti-immigration sentiment in North America [35, 55] is operating in subtler ways in the Canadian context rather than more overt discriminatory acts. Conversely, it is also plausible that the absence of an association between neighbourhood discrimination and cyber-victimization for immigrants reflects underlying differences in socialization processes related to time-since-arrival among immigrants [56]. This interpretation is consistent with prior research showing that recent immigrants are generally less likely to report discrimination than native-born counterparts and more established immigrants, the latter of whom may have different expectations of societal inclusion and perceptions of unequal treatment [57].

### Strengths and limitations

Our findings extend prior literature in several ways. First, our stratified approach allowed for interpretation of differences by immigrant status and sex, making it possible to examine comparisons that were not explored in previous studies. Second, our leveraging of a demographically and geographically dispersed weighted sample improved generalizability of findings to the entire country-level population. Third, our analysis expands consideration of cyber-victimization beyond youth to include older adults, a population whose experiences of the internet and social media are often overlooked. Lastly, we incorporated a broad social-ecological analytic approach, including neighbourhood factors, which attempted to respond to cautions raised in recent studies about the tendency for an overly narrow focus on individual and family-level factors explaining patterns of cyber-victimization.

Results, however, must also be interpreted within the limitations of our study. The study’s primary limitation is the cross-sectional study design and data, which implies the directionality of many associations found, are unknown. We were also not able to identify how associations may vary over time, especially for immigrants whose experiences and perspectives on what constitutes bullying, may change the longer they reside in the host country. Second, there was a lack of available variables related to our cyber-victimization outcome. For example, we could not know the types or sources (medium and perpetrators) of cyber-victimization in order to better understand this phenomenon across immigrant/non-immigrants and males/females. Third, we have only a crude measure of biological sex in available data and not a measure of gender, which precluded capturing various aspects of gender identity and how these may also intersect with our outcome. Additionally, English/French language ability for the GSS was an inclusion criterion, so the most marginalized immigrants, such as refugees, for whom forced migration has been associated with lower fluency of official language, are more likely to be excluded. In future, national-level data that are not limited by language ability are needed to better represent immigrants’ experiences.

### Future research

Our findings highlight several directions for future research in this area. Importantly, our main finding underscores the need to investigate the complex manner in which immigrant status and gender appear to place immigrant men at higher risk than women. Also, though we did not find an association between cyber-victimization and visible minority status, consistent with some prior research examining race and cyber-bullying [9, 26], we suspect this surprising result may reflect a limitation of the visible minority measure, which aggregates many different racial/ethnic groups. Future research should thus examine how race/ethnicity may contribute to risks for cyber-bullying, along with exploration of other social factors, such as migration context (i.e., refugee status, time since arrival, region, of origin), socioeconomic status, and sexual orientation. Additional investigation into how and for whom neighbourhood/place-based factors affect cyber-victimization risk, should also incorporate multilevel analyses, with objective measures of neighbourhoood characteristics and greater attention to how these may exacerbate or buffer risk. Lastly, a more complete study of cyber-victimization and immigrant status should examine the characteristics of perpetrators and the mediums used for bullying, including attention to how immigrants’ cyber-worlds may potentially also encompass cyber-victimization perpetrated by sources in countries of origin.

## Conclusions

This study expands knowledge about factors associated with cyber-victimization among immigrants and non-immigrants using, for the first time, a large, nationally-representative sample. Our main findings show that immigrants and non-immigrants experienced similar rates of cyber-victimization and that there was a notable increased vulnerability of males among immigrants that was not detected among non-immigrants. Increased vulnerability to cyber-victimization was also more pronounced among immigrants with a history of child maltreatment and those residing in an unwelcoming neighbourhood. These findings suggest that gender-stratified analyses should be a key avenue for future research on cyber-victimization among immigrants, and also demonstrate strong support for incorporating a broad social-ecological perspective to better elucidate relationships between immigrant status, gender and cyber-victimization.

## Data Availability

The data that support the findings of this study are available from Statistics Canada but restrictions apply to the availability of these data, which were used with approvals for the current study, and so are not publicly available.

## Abbreviations

aPOR: Adjusted prevalence odds ratio
95% CI: 95% confidence interval
GSS: General Social Survey
IPV: Intimate partner violence
